# Providing laypeople with results from dynamic infectious disease modelling studies affects their allocation preference for scarce medical resources—a factorial experiment

**DOI:** 10.1186/s12889-022-13000-7

**Published:** 2022-03-23

**Authors:** Nicole Rübsamen, Benno Garcia Voges, Stefanie Castell, Carolina Judith Klett-Tammen, Jérôme Oppliger, Pius Krütli, Timo Smieszek, Rafael Mikolajczyk, André Karch

**Affiliations:** 1 Institute of Epidemiology and Social Medicine, University of Münster, Albert-Schweitzer-Campus 1, 48149 Münster, Germany; 2 Department of Epidemiology, Helmholtz Centre for Infection Research (HZI), Braunschweig, Germany; 3 Department of Environmental Systems Science, ETH Zürich, Zürich, Switzerland; 4 MRC Centre for Outbreak Analysis and Modelling, Department of Infectious Disease Epidemiology, Imperial College School of Public Health, London, UK; 5 Modelling and Economics Unit, Statistics, Modelling, and Economics Department, Public Health England, London, UK; 6 Institute for Medical Epidemiology, Biometry, and Informatics (IMEBI), Medical Faculty of the Martin Luther University Halle-Wittenberg, Halle (Saale), Germany

**Keywords:** Allocation strategies, Factorial design, STI

## Abstract

**Background:**

Allocation of scarce medical resources can be based on different principles. It has not yet been investigated which allocation schemes are preferred by medical laypeople in a particular situation of medical scarcity like an emerging infectious disease and how the choices are affected by providing information about expected population-level effects of the allocation scheme based on modelling studies. We investigated the potential benefit of strategic communication of infectious disease modelling results.

**Methods:**

In a two-way factorial experiment (*n* = 878 participants), we investigated if prognosis of the disease or information about expected effects on mortality at population-level (based on dynamic infectious disease modelling studies) influenced the choice of preferred allocation schemes for prevention and treatment of an unspecified sexually transmitted infection. A qualitative analysis of the reasons for choosing specific allocation schemes supplements our results.

**Results:**

Presence of the factor “information about the population-level effects of the allocation scheme” substantially increased the probability of choosing a resource allocation system that minimized overall harm among the population, while prognosis did not affect allocation choices. The main reasons for choosing an allocation scheme differed among schemes, but did not differ among those who received additional model-based information on expected population-level effects and those who did not.

**Conclusions:**

Providing information on the expected population-level effects from dynamic infectious disease modelling studies resulted in a substantially different choice of allocation schemes. This finding supports the importance of incorporating model-based information in decision-making processes and communication strategies.

**Supplementary Information:**

The online version contains supplementary material available at 10.1186/s12889-022-13000-7.

## Background

Medical resources can be scarce because of restricted supply chains (e.g., vaccines) or high costs (e.g., antiretroviral therapy). Even in high-income countries, generally available resources can become scarce during emergencies like a pandemic. Persad et al. [1, 2] proposed guiding principles for the allocation of scarce resources: equal treatment (random selection, waiting list), utilitarianism (prognosis, number of lives saved), prioritarianism (sickest first, youngest first), and instrumental value (rewarding social usefulness in the past or in the future). Yousef et al. [3] added the two principles monetary contribution to the costs of one’s own medical treatment and individual behaviour (not engaging in risky behaviours that caused one’s medical condition) to this list.

The allocation of scarce treatment and prevention measures against infectious diseases is particularly challenging since the prevention or reduction of infectiousness directly affects the risk of infection for other individuals in the population. Dynamic infectious disease modelling studies can be used to assess the effects of different allocation schemes on a population level and to provide evidence for public health decision-making [4, 5]. An example of a compartmental model of disease dynamics was published by Mikolajczyk et al. [6] who showed how to estimate the number of deaths from influenza under different intervention strategies. Nevertheless, it is not clear if people consider such results from modelling studies as an important source of information. Furthermore, information from modelling studies underscores the utilitarian perspective, i.e., looking for the allocation scheme that saves most human lives or maximises (on average) a favourable outcome, while possibly ignoring the individual suffering. Zhang et al. [7] discuss this dilemma in a commentary about the consequences of redistributing antiretroviral therapy in low-resource settings to sicker HIV/AIDS patients, and not to patients immediately upon diagnosis, which would minimize overall harm among the population [1]. In addition, utilitarian allocation schemes regarding sexually transmitted infections (STI) often favour those individuals with the riskiest behaviour.

When confronted with hypothetical situations of scarcity, health professionals may choose different allocation schemes compared with laypeople [8]. Some authors have asked experts and laypeople to rank allocation schemes [3, 9], but it has not yet been investigated which allocation schemes are chosen by laypeople in the context of infectious disease prevention and how the choices are affected by providing information about expected results based on modelling studies. This is relevant for communication of decisions regarding allocation: If the allocation scheme by the authorities is not in line with the choices of the general population, this decreases the acceptance of their implementation [10]. By surveying a sample of inhabitants of Lower Saxony, Germany, we investigated the effect of providing information about expected population-level outcomes (based on dynamic infectious disease modelling studies) on the choice of allocation schemes. In addition, we investigated if prognosis of disease (time until death in case of infection) plays a role in choosing an allocation scheme. Our study aimed to provide insight for public health professionals to understanding the mind-set of the general population, as well as the potential benefit of strategic communication of infectious disease modelling results.

## Methods

### Sample

We implemented a factorial experiment within HaBIDS, an online panel to assess preventive behaviour regarding infectious diseases [11, 12]. In brief, 26,895 individuals (15–69 years old) from four districts in Lower Saxony, Germany were invited, of which 9% participated in the panel. In February 2016, the questionnaire about allocation of scarce medical resources (Additional File 1) was activated; 1,037 participants were still enrolled in HaBIDS at that time.

Of those, 878 individuals participated in this factorial experiment. Compared with the sampling frame (the four districts in Lower Saxony), the participants of this study were older (median age group 50–54 years vs. 40–44 years in the sampling frame), more likely to be female (59.9% vs. 50.0%), more likely to have a university degree (41.8% vs. 13.5%), and more likely to be married (59.0% vs. 46.7%).

### Factorial experiment

Participants were presented with a hypothetical resource allocation problem concerning an STI that is spreading in a city. Participants were 1:1 randomly assigned to a scenario where either prevention (vaccination) or treatment (cure) should be distributed.

**Fig. 1:**
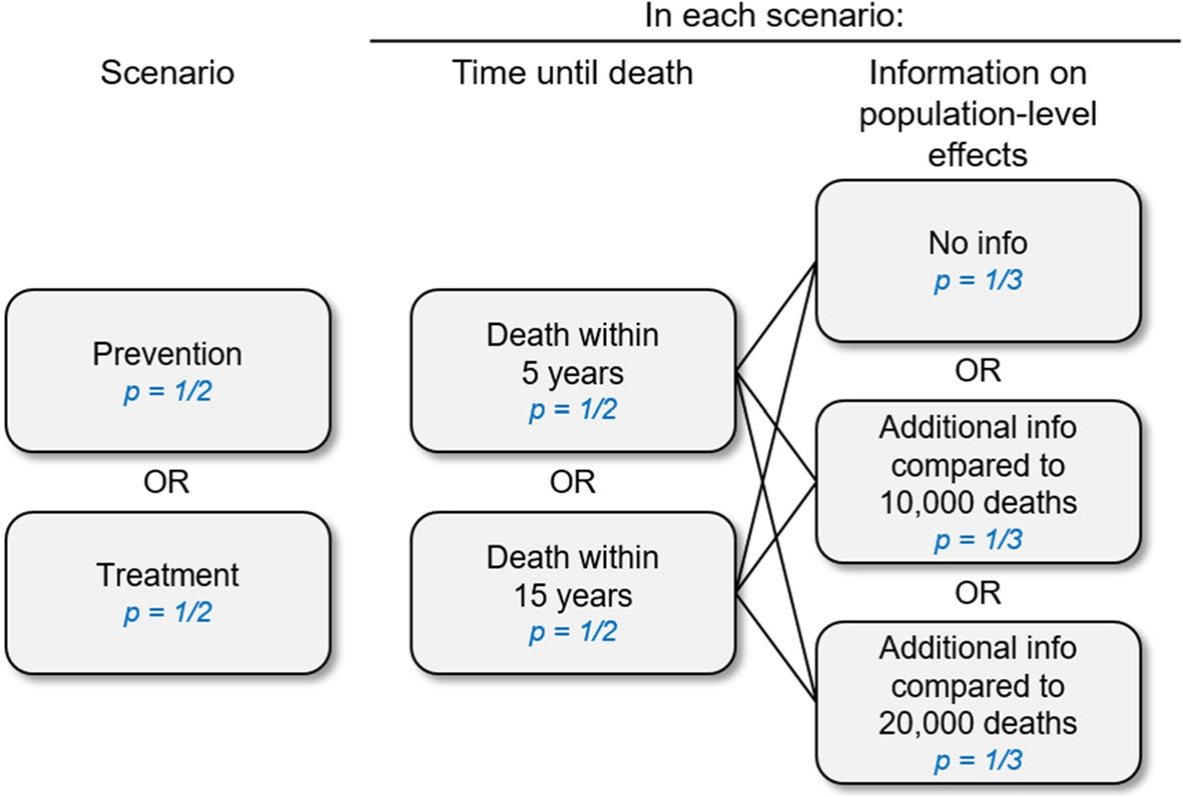
Factorial design

The description stated that inhabitants differ in how often they change sex partners and how often they have several sex partners at the same time (information corresponding to results from the Natsal study [13]). Participants were randomly assigned to one of 6 combinations, based on a 2 × 3 factorial design (Fig. 1): time until death as indicator of severity of the disease (5 years vs. 15 years) × model-based information on expected population-level effects of each allocation scheme (number of the avoided deaths in two versions as described below vs. no information). The two factors were randomized independently from each other.

Participants were asked to choose among the following options for allocation: “random allocation” (i.e., equal treatment), “young individuals first” (i.e., prioritarianism), “promiscuous individuals first” (i.e., utilitarianism in the case of this STI), “individuals with long-lasting partnerships first” (i.e., individual behaviour), or “undecided”. In the scenario “treatment”, an additional option “first come, first served” was given because the waiting-list principle is often applied to (expensive) treatments, but usually not to (non-expensive) vaccinations. We excluded allocation schemes based on instrumental value or monetary contribution because these are not applicable to STI.

**Fig. 2:**
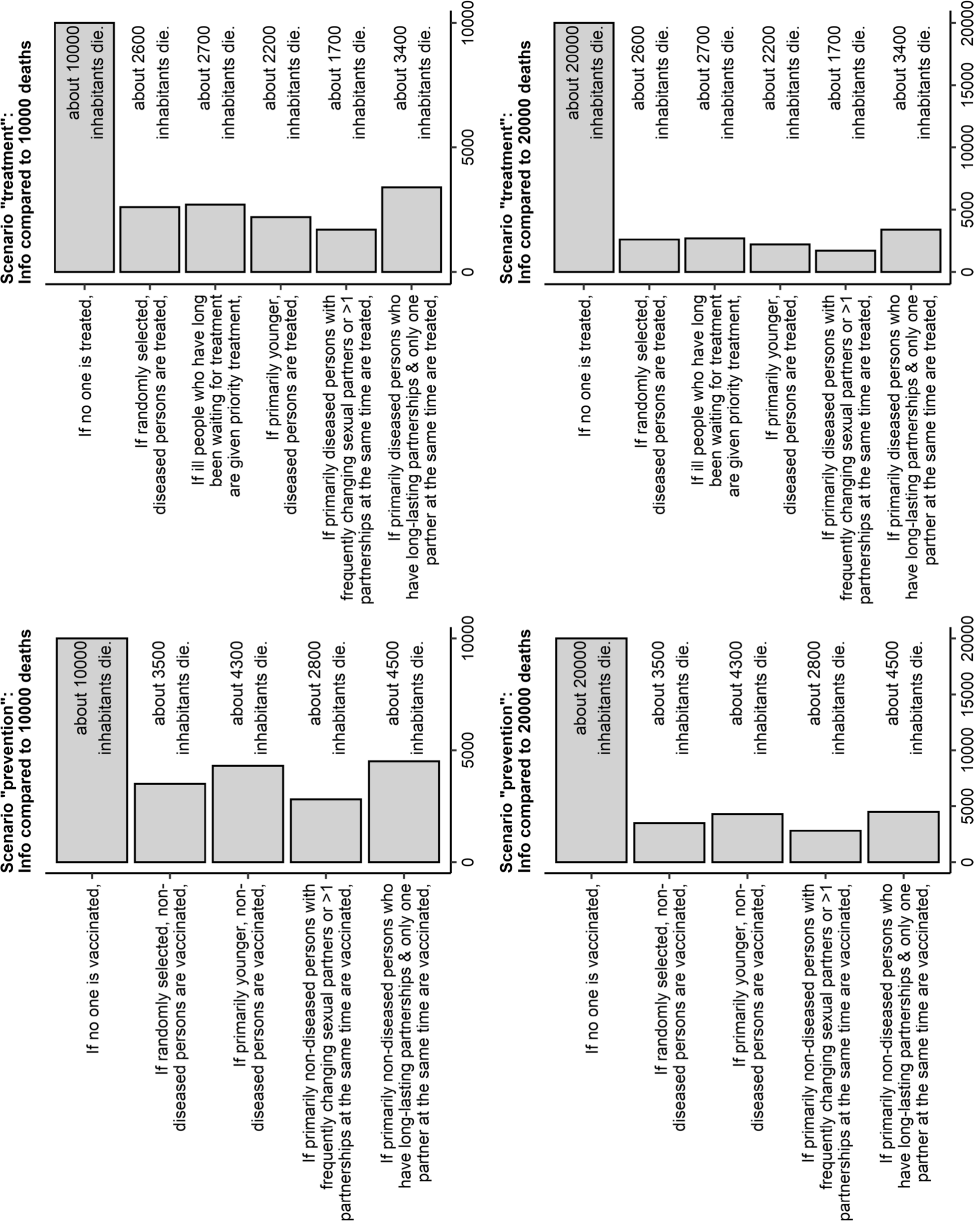
Versions of the model-based information on expected population-level effects

For the model-based information on expected population-level effects, there were three options: no information, or one of two versions of information on the effects of the various allocation schemes. The information consisted of a bar chart (Fig. 2): The top bar showed the expected number of deaths in the absence of treatment. Then, for each allocation scheme, a bar showed the expected number of deaths if the treatment was distributed according to this scheme. The expected numbers of deaths were based on the results of a simple compartmental model of the disease dynamics, similar to the model published by Mikolajczyk et al. [6]. The model-based information on expected population-level effects was prepared before starting the experiment; participants were presented with the static bar charts (Fig. 2) during the experiment. There was no interaction between the dynamic infectious disease model and the experiment, i.e. the participants’ choices of allocation schemes did not alter model-based information on expected population-level effects.

The two versions of the information differed only in the top bars of the bar chart: One version showed that in the absence of treatment, 10,000 inhabitants would die while the other stated that 20,000 inhabitants would die (Additional File 1). All other numbers of deaths (i.e., all other bars) were equal for both versions, so that in the version with 10,000 deaths the relative differences between various allocation schemes appeared substantially larger, while in the version with 20,000 deaths the various allocation schemes appeared more similar to each other (but more different from the scenario with no treatment).

### Sample size

We aimed to investigate any difference in the distribution of the choices of allocation schemes dependent on the randomization factors. We estimated that 500 participants per scenario (i.e., 1,000 participants in total, corresponding to the size of the HaBIDS panel), would allow us to achieve at least 95% power in a chi-squared test if there was a medium or large effect (Cohen’s effect size index [14] w = 0.3 or w = 0.5, respectively).

The 1:1 randomization resulted in 441 participants in the scenario “prevention” (Additional File 2) and 437 participants in the scenario “treatment” (Additional File 3).

### Statistical analysis

First, we investigated the influence of the scenario (prevention vs. treatment) on the choice of allocation scheme with Pearson’s chi-squared test. We repeated this test in the subsets of participants who received either no information or any additional information.

The following statistical tests were conducted within each scenario separately: The influence of each factor (model-based information and disease severity) on the choice of allocation scheme was investigated with Pearson’s chi-squared tests. The factor “model-based information on expected population-level effects” was entered as no information vs. any additional information for the primary analysis. To assess if there was any interaction between the two factors, we used the likelihood-ratio test to compare a multinomial log-linear model including the interaction “any info × severity” with a model including the two main factors only (R package ‘nnet’ [15] version 7.3–16). In the same way, we investigated if there was an interaction “any info × university degree” to exclude that education affected how individuals processed model-based information. Because university degree was not randomized, but a sociodemographic characteristic of the participants, we included age and sex in the multinomial log-linear models as proposed by VanderWeele and Knol [16]. We used the likelihood-ratio test to compare the model *multinom(Choice of allocation scheme* ~ *age* + *sex* + *any info* + *university degree)* to *multinom(Choice of allocation scheme* ~ *age* + *sex* + *any info* + *university degree* + *any info* × *university degree)*.

To investigate if the number of deaths in the event of no treatment influences the choice of allocation scheme by making the differences between them appear larger or smaller, the two versions of the factor “model-based information on expected population-level effects” were analysed with chi-squared tests among the participants who had received any additional information. All analyses were performed with R [17] version 4.1.2.

### Qualitative analysis

In a free text field, participants were also asked to give the reason for their choice of allocation scheme within the factorial experiment. These responses were evaluated with a modified and extended structured content analysis according to Mayring [18]. They were presented to three independent researchers who developed a category system with subcategories, which was further elaborated with the help of an external researcher. Three researchers, one of whom was not involved in the previous process, applied the category system independently. Intercoder reliability was calculated by using Krippendorff’s alpha [19], which indicates the overall match of the three encoders (0 = no match, 1 = perfect match). A consensus was found for any nonmatching categorizations according to pre-established rules by two researchers to determine the final classification. The structured content analysis revealed four categories with a total of eight subcategories (Additional File 4). Krippendorff’s alpha among the three encoders was lowest for “condemnation of a particular lifestyle” (0.51) and highest for “minimize risks/number of deaths” (0.83).

## Results

When comparing the two scenarios “treatment” versus “prevention,” the frequency of undecided participants was higher in the scenario “treatment” (28.4%) than in the scenario “prevention” (16.6%), while the frequency of choosing the utilitarian allocation scheme was lower (24.3% vs. 46.0% in the scenario “treatment” vs. “prevention,” respectively). There was evidence for an influence of the scenario on the choice of allocation scheme (*p* < 0.001). This scenario effect was also present in the subgroups of participants with or without model-based information (Table 1).

**Table 1:** Relative frequencies (%) of choice of allocation scheme by randomization factors

	**Overall** (p_chi-squared test_ < 0.001)		**Model-based information** **on expected population-level effects**				**Time until death**			
	Prevention (*n* = 441)	Treatment (*n* = 437)	Prevention (p_chi-squared test_ < 0.001)		Treatment (p_chi-squared test_ = 0.004)		Prevention (p_chi-squared test_ = 0.77)		Treatment (p_chi-squared test_ = 0.82)	
			No Info^a^ (*n* = 149)	Additional Info^b^ (*n* = 292)	No info^a^ (*n* = 144)	Additional info^b^ (*n* = 293)	Death within 5 years (*n* = 227)	Death within 15 years (*n* = 214)	Death within 5 years (*n* = 231)	Death within 15 years (*n* = 206)
Random allocation (equal treatment)	12.2	6.6	12.8	12.0	8.3	5.8	12.8	11.7	6.1	7.3
First come, first served (waiting-list)	NA	22	NA	NA	27.1	19.5	NA	NA	19.9	24.3
Young individuals first (prioritarianism)	12.5	9.2	22.1	7.5	11.1	8.2	13.7	11.2	10.0	8.3
Promiscuous individuals first (utilitarianism)	46	24.3	34.2	52.1	12.5	30.0	45.4	46.7	26.0	22.3
Long-lasting partnerships first (individual behaviour)	12.7	9.6	16.8	10.6	9.7	9.6	11.0	14.5	10.0	9.2
Undecided	16.6	28.4	14.1	17.8	31.2	27.0	17.2	15.9	28.1	28.6

^a^p_chi-squared test_ < 0.001 for prevention vs. treatment among participants who did not receive any information

^b^p_chi-squared test_ < 0.001 for prevention vs. treatment among participants who received any information

Chi-squared tests indicated an effect of the factor “model-based information on expected population-level effects” on the choice of an allocation scheme (Table 1). The frequency of choosing the utilitarian allocation scheme was higher among participants who received information about expected effects of the various choices based on modelling (52.1% vs. 34.2%, *p* < 0.001, in the scenario “prevention” and 30.0% vs. 12.5%, *p* = 0.004, in the scenario “treatment”). The frequencies of choosing random allocation or “undecided” were not affected by providing additional information. The higher frequency of choosing the utilitarian allocation scheme in the scenario “prevention” was accompanied by lower frequencies of choosing “young individuals first” and “long-lasting partnerships first”. This shift was not that pronounced in the scenario “treatment”.

Differences in the population-level effects (resulting from the number of deaths in the absence of treatment) played no role for the choice of the utilitarian allocation scheme among participants who had received model-based information on expected population-level effects; in the scenario “prevention”, 54.9% chose the utilitarian allocation scheme among those presented with 10,000 deaths in the absence of treatment compared with 49.3% among those presented with 20,000 deaths (Additional File 5). In the scenario “treatment”, 28.1% vs. 32.0% chose the utilitarian allocation scheme among those presented with 10,000 vs. 20,000 deaths in the absence of treatment (Additional File 5).

There was neither evidence for an effect of the factor “time until death” on choosing an allocation scheme in the scenario “prevention” (e.g., utilitarian scheme: 45.4% for 5 years until death vs. 46.7% for 15 years until death, *p*-value of chi-squared test across all categories = 0.77; Table 1) nor in the scenario “treatment” (e.g., utilitarian scheme: 26.0% vs. 22.3%, *p* = 0.82). The distribution of chosen allocation schemes did not differ when stratifying by both “time until death” and “model-based information on expected population-level effects” (Additional File 6) except for the scenario “prevention” where a longer time to death doubled the percentage of participants who chose “individual behaviour” if there was no additional information (11.5% for 5 years until death vs. 22.5% for 15 years until death). This effect was not seen in participants who received additional information nor in the scenario “treatment” (Additional File 6). In likelihood-ratio tests between two nested multinomial log-linear models, there was no evidence that both randomization factors interacted with each other (*p* = 0.59 and *p* = 0.52 in the scenario “prevention” and “treatment”, respectively). There was also no evidence for the interaction “any info × university degree” (*p* = 0.41 and *p* = 0.07 in the scenario “prevention” and “treatment”, respectively; Additional File 7), although it seemed that in the scenario “treatment”, providing additional information made participants with university degree choose the utilitarian scheme more often (10.2% without information vs. 35.4% with information) and “individual behaviour” less often (13.6% vs. 6.3%), while additional information made participants without university degree choose “individual behaviour” more often (7.8% vs. 11.9%) and “equal treatment” less often (10.4% vs. 5.0%). It has to be noted that the percentage of participants with university degree was higher among men than that among women (49.5% vs. 37.2%) in the scenario “treatment”.

### Qualitative analysis

**Fig. 3:**
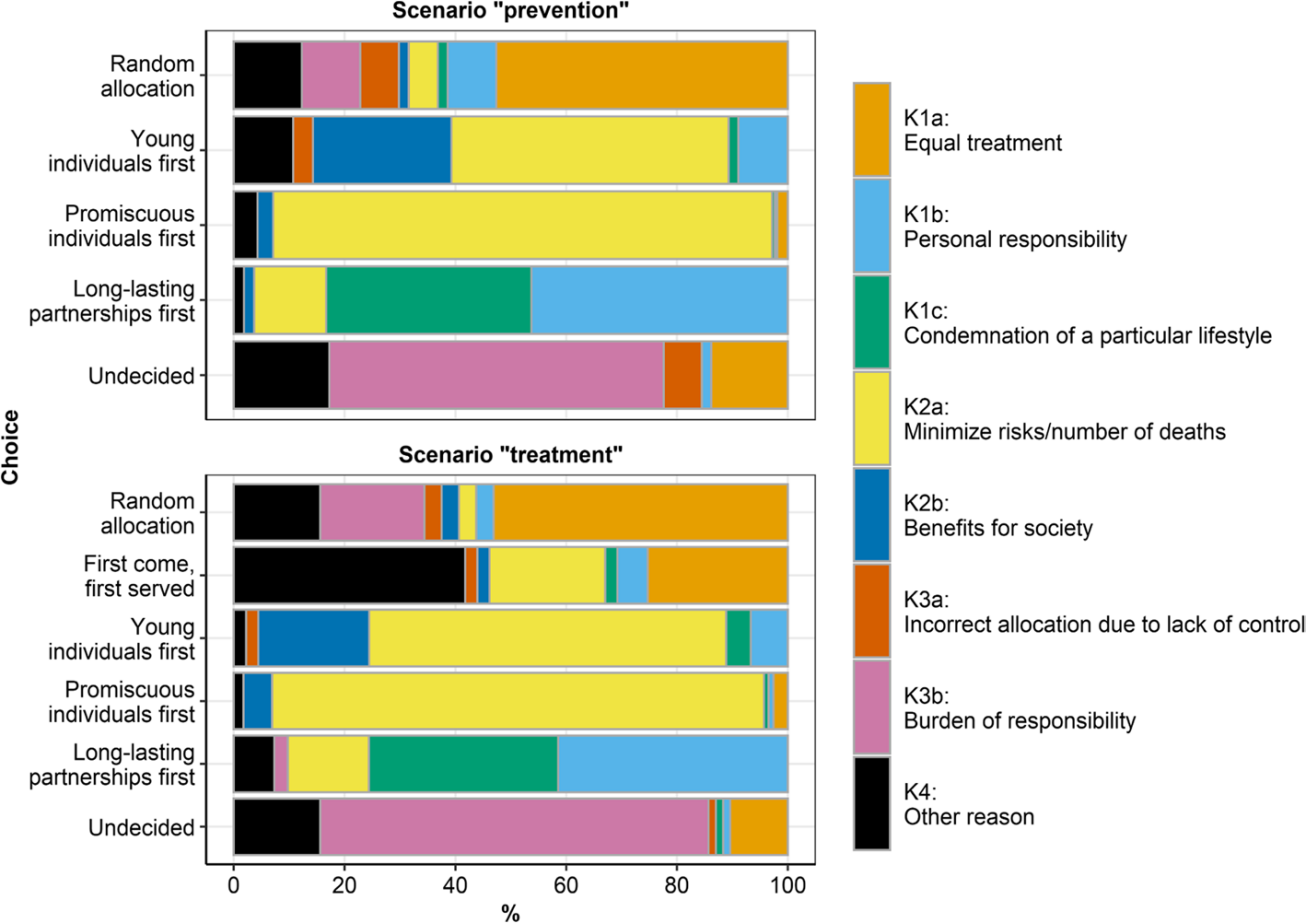
Reasons for choosing the allocation schemes

Eighty-three percent of the participants (*n* = 728) entered reasons for their choice of an allocation scheme. Utilitarian choices were provided by 63.4% of these participants in the scenario “prevention” and 47.8% in the scenario “treatment” (Additional File 4). The main reasons for choosing an allocation scheme differed among all the schemes (Fig. 3). The percentage of participants who chose “promiscuous individuals first” to “minimize risks/number of deaths” was above 90%. This did not differ among those who received additional model-based information on expected population-level effects and those who received no additional information.

## Discussion

We show that providing information about expected population-level outcomes (based on dynamic infectious disease modelling studies) affects the choice of preferred allocation schemes among medical laypeople.

If prevention measures had to be distributed, individuals predominantly chose the allocation scheme that minimized the number of deaths, followed by allocation schemes that correspond to the “need” principle [20]. This trend towards the utilitarian allocation scheme accentuated if additional information about the population-level effect was provided. If treatment had to be distributed, most individuals selected “undecided”, followed by the utilitarian allocation scheme and waiting-list. If additional information about the population-level effect was provided, the frequency of choosing the utilitarian allocation scheme doubled so that one-third of all participants chose to prioritize promiscuous individuals.

Our results show that individuals differentiate between prevention and treatment; without additional information, prevention is allocated to the group with risky behaviour, but treatment is not. There seems to be a sound understanding of the transmission of infections in the population, and individuals are willing to allocate vaccinations to the people that contribute most to transmission, irrespective of their lifestyle. This is not surprising as this option shows the best effect at a societal level.

If, however, people have been infected because of their lifestyle, only a small proportion of individuals would allocate treatment to these people. Additional information about the expected number of deaths could persuade individuals to allocate treatment to infected promiscuous people. The qualitative analysis revealed that individuals attribute the number of deaths to the whole population in the scenario “prevention”, but only to the group of promiscuous people in the scenario “treatment.” This can be interpreted as retaliation of those self-responsible for their medical emergency. It is known from earlier studies that people tend to withdraw aid from individuals being self-responsible for their precarious situation [21] even if it is at costs of their benefits [22].

Our results provide insight for public health professionals to understanding the mind-set of the general population. We do not postulate that resource allocation by authorities should rely on the preferred allocation schemes of laypeople; instead, scientific reasoning (incl. information on the expected population-level effects from dynamic infectious disease modelling studies) should guide any public health decision-making. For authorities, it is important to understand population’s preferences and gain their trust in governmental strategies [23]. An example of strategic communication of infectious disease modelling results during the Covid-19 pandemic was the “flattening the curve” approach, which meant slowing the rate of infection and reducing the number of cases so that it did not overburden the healthcare system [24, 25]. Studies about population’s preferences have also been implemented during the Covid-19 pandemic, e.g. concerning exit strategies from lockdown in Germany [26]. While the latter study did not provide participants with information on the expected population-level effects of their choice, there is, to our knowledge, no study so far that investigated acceptance of the “flattening the curve” approach [27]. Modifications of our experiment could be applied to investigate such open questions regarding Covid-19 and other infectious diseases.

Our study provides hints that preferences might differ in subgroups of the population (e.g., based on education) or dependent on the characteristics of the disease (e.g., time until death). Further research is needed to confirm these findings and to disentangle confounding (e.g., correlation of male sex and university degree).

### Limitations

There may be implicit and explicit content overlaps among the studied allocation schemes. It was stated in the survey that younger inhabitants change their sex partners more often than older ones. This could have created logical overlaps between the options “young individuals first” and “promiscuous individuals first,” but such overlaps cannot be avoided in a real-life scenario. While we could not assess whether our sample was representative for Germany concerning attitudes towards STI and promiscuity, our study population contained people with more than average education indicating that the results are probably not generalizable to the general population. We, however, did not find evidence that education affected how individuals processed model-based information in our sample.

## Conclusions

Providing information on the expected population-level effects from dynamic infectious disease modelling studies resulted in a substantially different choice of preferred allocation schemes. Strategic communication of infectious disease modelling results, e.g. providing laypeople with more information about the expected death toll when implementing infection control measures compared to not implementing them, can help to increase acceptance of these measures.

## Supplementary Information

**Additional file 1. **English translation of the German questionnaire.

**Additional file 2.** Baseline demographic characteristics for each group within the scenario “prevention”.

**Additional file 3.** Baseline demographic characteristics for each group within the scenario “treatment”.

**Additional file 4.** Result of the structured content analysis.

**Additional file 5.** Relative frequencies (%) of choice of allocation scheme in the two scenarios stratified by the randomization factor “model-based information on expected population-level effects”.

**Additional file 6.** Relative frequencies (%) of choice of allocation scheme in the two scenarios stratified by the randomization factors “time until death” and “model-based information on expected population-level effects”.

**Additional file 7.** Relative frequencies (%) of choice of allocation scheme in the two scenarios stratified by highest completed educational level and the randomization factor “model-based information on expected population-level effects”.

## Data Availability

The datasets generated and/or analysed during the current study are available from the corresponding author on reasonable request.

## Abbreviations

HaBIDS: Hygiene and Behaviour Infectious Diseases Study
STI: Sexually transmitted infection
