# Supplementary material for: Providing laypeople with results from dynamic infectious disease modelling studies affects their allocation preference for scarce medical resources—a factorial experiment
Source: BMC Public Health. 2022 Mar 23;22:572. doi: 10.1186/s12889-022-13000-7 (PMC8940588; doi:10.1186/s12889-022-13000-7)
Supplement: Supplementary file 1 — Additional file 1. English translation of the German questionnaire. [file 12889_2022_13000_MOESM1_ESM.pdf]

Additional File 1 – English translation of the German questionnaire
(English version not validated for use in surveys)

Table of contents

Allocation of scarce medical resources 2
Randomisation (not visible for the participants) 2
Distribution problem (▼ FM_Szenario == "1") 2
Distribution problem (▼ FM_Szenario == "2") 4
Question about distribution problem 6

▼ indicates that the question is displayed only if the respective condition is met.
[] Variable names are written in square brackets. They are not visible for the participants.

Allocation of scarce medical resources

In medicine, medical resources may not be available to all who need them. One example is organ transplantation. In the case of a flu pandemic, the vaccine can become scarce. The purpose of this questionnaire is to examine how scarce medical resources should be distributed. In the following questionnaire, there are no "right" or "wrong" answers. We are much more interested in your personal opinion about the distribution of medical resources.

Randomisation (not visible for the participants)

[FM_Szenario]
random number (1 or 2)

[FM_Mortalitaet]
random number (0 or 1)

[FM_Info]
random number (0 or 1 or 2)

[FM_Fragestellung]
random number (0 or 1)

Distribution problem (▼ FM_Szenario == "1")

▼ FM_Szenario == "1"
The following situation is invented. However, it shows similarities with processes in reality. In a city, a sexually transmitted disease has spread for 20 years. This disease leads to death within if (FM_Mortalitaet == "1", "15 years", "5 years"). Currently every 50th inhabitant of the city is ill. The inhabitants differ in how often they change sexual partners and how often they have several sexual partners at the same time: approximately every 5th inhabitant belongs to the group with frequently changing partners or several partners at the same time. The remaining 80% of the population rarely change sexual partners. Younger residents of this city are changing their partners more often than older ones. A vaccine is now available for the first time. This protects reliably against contamination; already diseased persons are not cured by the vaccination however. The vaccine stock is currently not sufficient for all residents of the city. The vaccination can be distributed according to different criteria.

▼ (FM_Szenario == "1") AND ((FM_Info == "1") OR (FM_Info == "2"))
Without vaccination if (FM_Info == "1", "10,000", "20,000") inhabitants of the city would die of the disease. Researchers have estimated how much the number of deaths can be reduced depending on how the vaccination is distributed.

▼ FM_Info == "1"

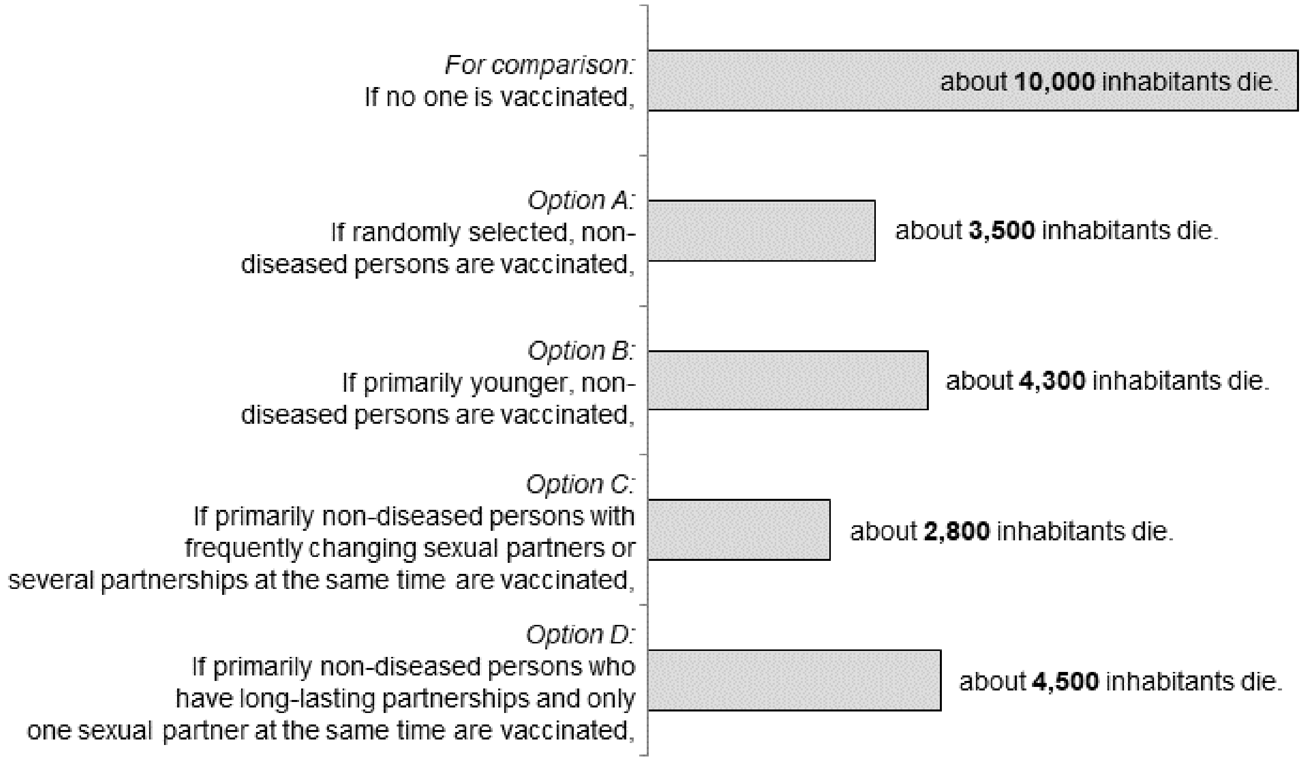
For comparison:
If no one is vaccinated,
about 10,000 inhabitants die.
Option A:
If randomly selected, non-diseased persons are vaccinated,
about 3,500 inhabitants die.
Option B:
If primarily younger, non-diseased persons are vaccinated,
about 4,300 inhabitants die.
Option C:
If primarily non-diseased persons with frequently changing sexual partners or several partnerships at the same time are vaccinated,
about 2,800 inhabitants die.
Option D:
If primarily non-diseased persons who have long-lasting partnerships and only one sexual partner at the same time are vaccinated,
about 4,500 inhabitants die.

▼ FM_Info == "2"

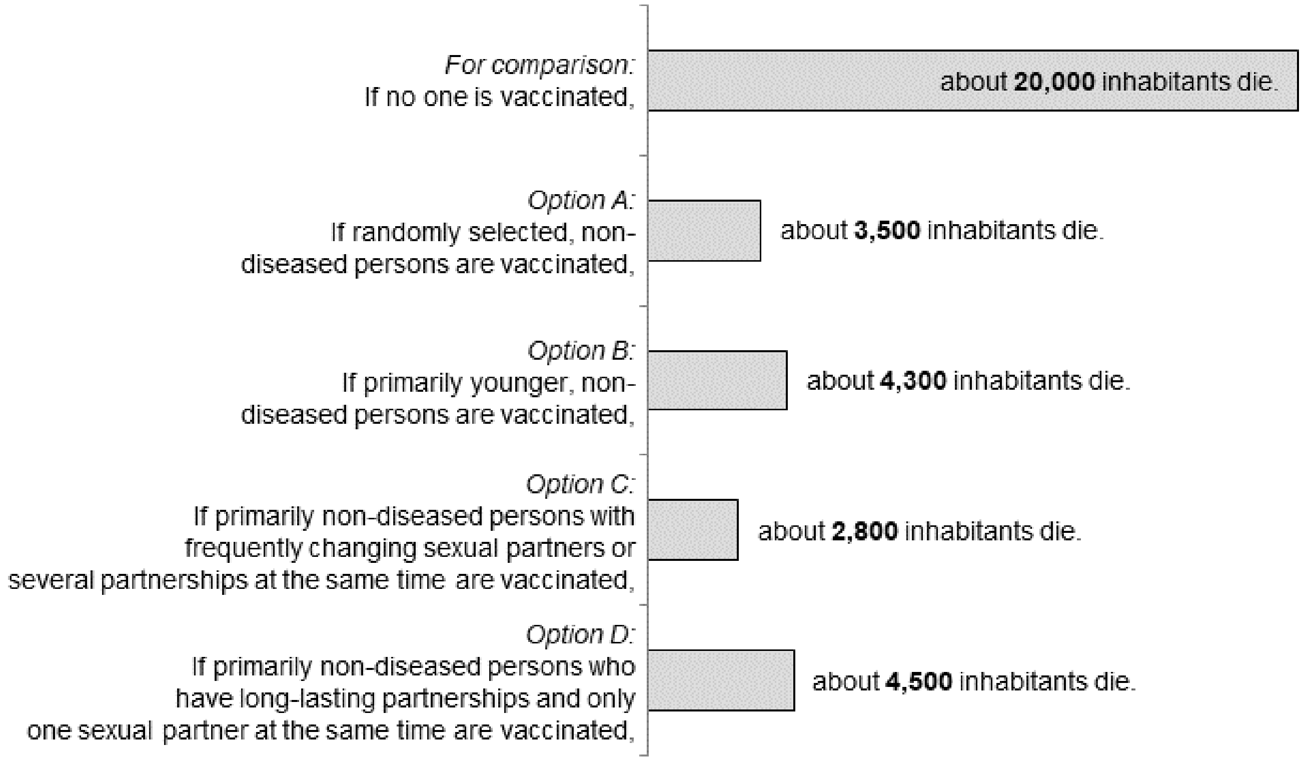
For comparison:
If no one is vaccinated,
about 20,000 inhabitants die.
Option A:
If randomly selected, non-diseased persons are vaccinated,
about 3,500 inhabitants die.
Option B:
If primarily younger, non-diseased persons are vaccinated,
about 4,300 inhabitants die.
Option C:
If primarily non-diseased persons with frequently changing sexual partners or several partnerships at the same time are vaccinated,
about 2,800 inhabitants die.
Option D:
If primarily non-diseased persons who have long-lasting partnerships and only one sexual partner at the same time are vaccinated,
about 4,500 inhabitants die.

Distribution problem (▼ FM_Szenario == "2")

▼ FM_Szenario == "2"
The following situation is invented. However, it shows similarities with processes in reality. In a city, a sexually transmitted disease has spread for 20 years. This disease leads to death within if (FM_Mortalitaet == "1", "15 years", "5 years"). Currently every 50th inhabitant of the city is ill. The inhabitants differ in how often they change sexual partners and how often they have several sexual partners at the same time: approximately every 5th inhabitant belongs to the group with frequently changing partners or several partners at the same time. The remaining 80% of the population rarely change sexual partners. Younger residents of this city are changing their partners more often than older ones. A treatment option is now available for the first time. An ill person who is treated can become as old as a healthy person. The treatment protects others from infection. The treatment is very expensive. Therefore, it is not possible to treat all persons who have been infected. The treatment can be distributed according to different criteria.

▼ (FM_Szenario == "2") AND ((FM_Info == "1") OR (FM_Info == "2"))
Without treatment, if (FM_Info == "1", "10,000", "20,000") residents of the city would die from the disease. Researchers have estimated how much the number of deaths can be reduced depending on how the treatment is distributed.

▼ FM_Info == "1"

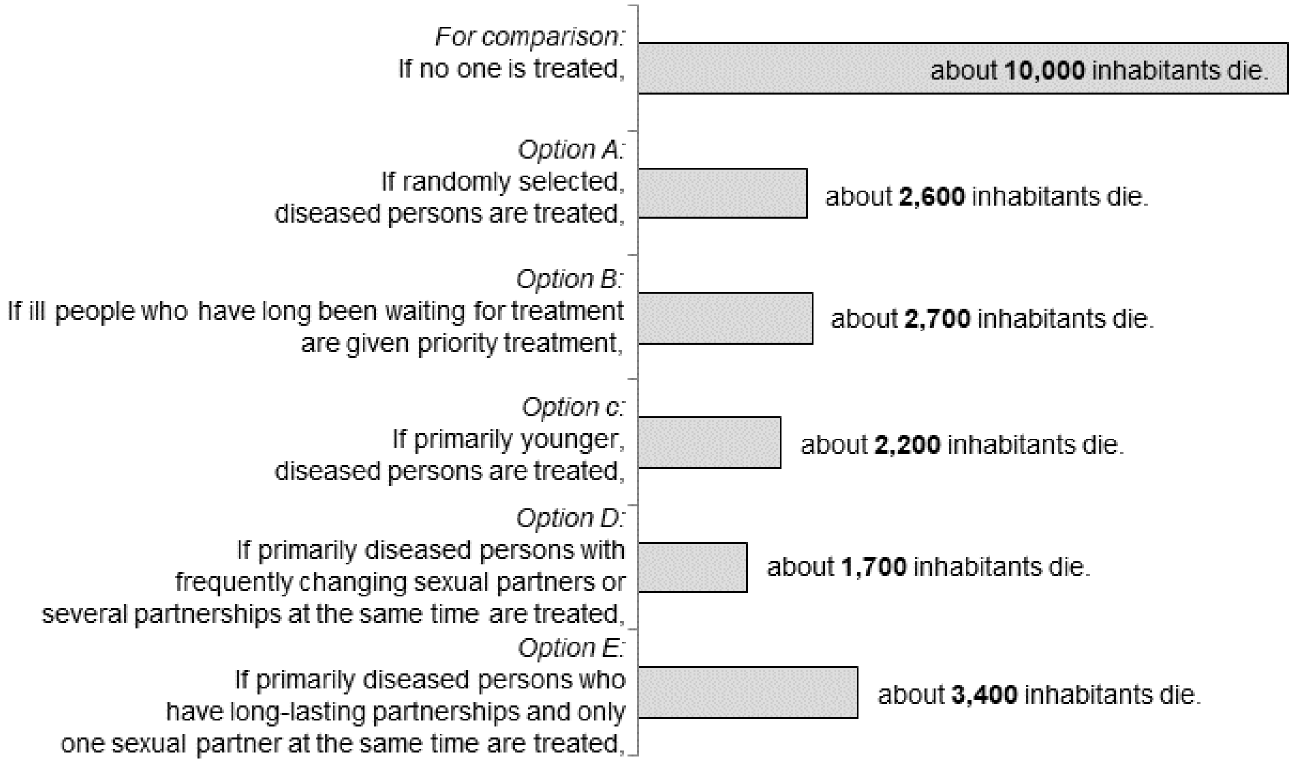

▼ FM_Info == "2"

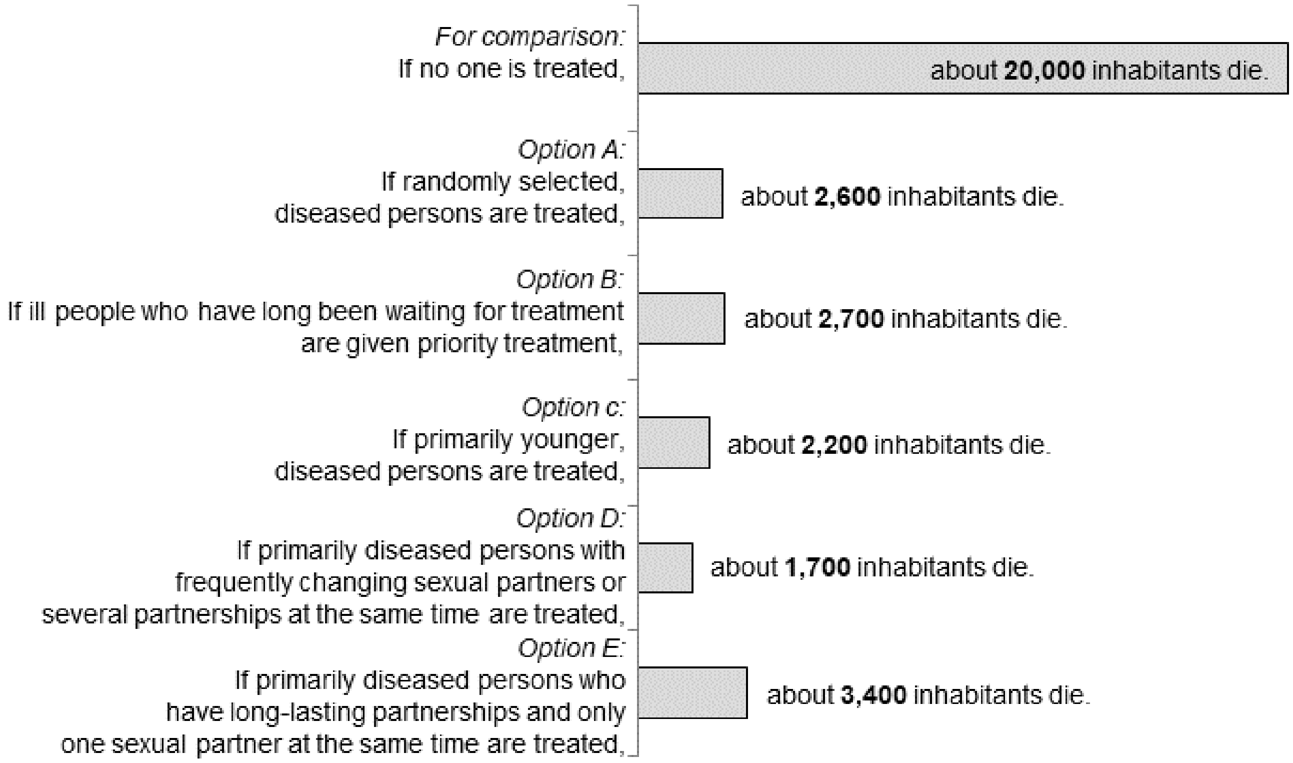
For comparison:
If no one is treated,
about 20,000 inhabitants die.
Option A:
If randomly selected,
diseased persons are treated,
about 2,600 inhabitants die.
Option B:
If ill people who have long been waiting for treatment
are given priority treatment,
about 2,700 inhabitants die.
Option c:
If primarily younger,
diseased persons are treated,
about 2,200 inhabitants die.
Option D:
If primarily diseased persons with
frequently changing sexual partners or
several partnerships at the same time are treated,
about 1,700 inhabitants die.
Option E:
If primarily diseased persons who
have long-lasting partnerships and only
one sexual partner at the same time are treated,
about 3,400 inhabitants die.

Question about distribution problem

[FM_Impfung_choice] if(FM_Fragestellung == "1", "How do you think the vaccinations should be distributed in the described situation?", "Which of the following distribution rules do you think is the fairest in the situation described?")

▼ FM_Szenario == "1"

- ☐ Option A: Randomly selected, non-diseased persons are vaccinated.
- ☐ Option B: Younger, non-diseased persons are primarily vaccinated.
- ☐ Option C: Priority is given to non-diseased individuals who often have changing sexual partners or several partnerships at the same time.
- ☐ Option D: Priority is given to non-diseased persons who have long-lasting partnerships and have only one sexual partner at the same time.
- ☐ I cannot decide.

[FM_Impfung_reason] if(FM_Impfung_choice == "90"," Why are you unable to decide? ", If (FM_Fragestellung ==" 1 "," Why did you choose this distribution of vaccination? "," Why do you consider the distribution rule you have chosen as the fairest? ")
Please justify your choice with 1 to 5 short sentences. Your answers are very important to the study.
enter your answer here

[FM_Behandlung_choice] if(FM_Fragestellung == "1", " How, in your opinion, should the treatment be distributed in the described situation? "," Which of the following distribution rules do you think is the fairest in the situation described? ")

▼ FM_Szenario == "2"

- ☐ Option A: Randomy selected ill persons are treated.
- ☐ Option B: Priority is given to ill people who have been waiting for treatment for a long time.
- ☐ Option C: Most younger, ill people are treated.
- ☐ Option D: Priority is given to ill-treated persons who frequently have changing sexual partners or several partnerships at the same time.
- ☐ Option E: Mainly ill people are treated who have long-lasting partnerships and have only one sexual partner at the same time.
- ☐ I cannot decide.

[FM_Behandlung_reason] if(FM_Impfung_choice == "90", "Why are you unable to decide?", If (FM_Fragestellung == "1", "Why did you choose this distribution of treatment?", "Why do you consider the distribution rule you have chosen as the fairest?")
Please justify your choice with 1 to 5 short sentences. Your answers are very important to the study.
enter your answer here
