# Supplementary material for: Providing laypeople with results from dynamic infectious disease modelling studies affects their allocation preference for scarce medical resources—a factorial experiment
Source: BMC Public Health. 2022 Mar 23;22:572. doi: 10.1186/s12889-022-13000-7 (PMC8940588; doi:10.1186/s12889-022-13000-7)
Supplement: Supplementary file 2 — Additional file 2. Baseline demographic characteristics for each group within the scenario “prevention”. [file 12889_2022_13000_MOESM2_ESM.pdf]

Additional File 2 – Baseline demographic characteristics for each group within the scenario "prevention"

Data are medians (25th percentile, 75th percentile) or numbers (%)

		Time until death		Information on population-level effects		
	**All (n = 441)**	**Death within 5 years (n = 227)**	**Death within 15 years (n = 214)**	**No info (n = 149)**	**Additional info compared to 10,000 (n = 144)**	**Additional info compared to 20,000 (n = 148)**
Age (years)	51.0 (38.4, 58.0)	50.5 (41.9, 58.0)	51.0 (37.9, 59.0)	52.0 (42.0, 58.0)	48.0 (36.4, 58.0)	51.0 (38.2, 59.0)
Female	287 (65.2)	142 (62.8)	145 (67.8)	101 (67.8)	88 (61.1)	98 (66.7)
**Highest completed educational level**						
Lower secondary education or apprenticeship	121 (28.0)	58 (26.1)	63 (30.0)	44 (29.7)	36 (25.5)	41 (28.7)
Still at upper secondary school	7 (1.6)	4 (1.8)	3 (1.4)	0 (0)	5 (3.5)	2 (1.4)
University entrance qualification	123 (28.5)	68 (30.6)	55 (26.2)	46 (31.1)	35 (24.8)	42 (29.4)
University degree	181 (41.9)	92 (41.4)	89 (42.4)	58 (39.2)	65 (46.1)	58 (40.6)
**Marital status**						
Married	250 (58.1)	134 (60.9)	116 (55.2)	93 (63.3)	74 (52.5)	83 (58.5)
Unmarried	128 (29.8)	58 (26.4)	70 (33.3)	40 (27.2)	47 (33.3)	41 (28.9)
Divorced	40 (9.3)	24 (10.9)	16 (7.6)	10 (6.8)	16 (11.3)	14 (9.9)
Widowed	12 (2.8)	4 (1.8)	8 (3.8)	4 (2.7)	4 (2.8)	4 (2.8)
