# Supplementary material for: Providing laypeople with results from dynamic infectious disease modelling studies affects their allocation preference for scarce medical resources—a factorial experiment
Source: BMC Public Health. 2022 Mar 23;22:572. doi: 10.1186/s12889-022-13000-7 (PMC8940588; doi:10.1186/s12889-022-13000-7)
Supplement: Supplementary file 4 — Additional file 4. Result of the structured content analysis. [file 12889_2022_13000_MOESM4_ESM.pdf]

Additional File 4 – Result of the structured content analysis

Category	Example	% in scenario "prevention" * (n = 383)	% in scenario "treatment" * (n = 345)
**1. Justice – stigma vs. personal responsibility**		24.8	26.6
**a. Equal treatment**	• "I don't want to play younger people off against older people, nor do I want to play sick people off against healthy people."	11.0	14.8
**b. Personal responsibility**	• "People must also do something for their health and therefore make conscious decisions in order to maintain their own health."	9.7	8.1
**c. Condemnation of a particular lifestyle**	• "Priority to decency and morality"<br>• "Healthy lifestyle has to be rewarded"	6.0	5.8
**2. Utilitarianism**		63.4	47.8
**a. Minimize risks/number of deaths or preserve years of life/number of people**	• "Because then very few people die"<br>• "Because that's how most people survive"	59.5	45.8
**b. Benefits for society**	• "Mr. Spock: the well-being of many is more important than the well-being of the individual"<br>• "And if you look at it economically, a young person is a fresh worker."	5.7	5.2
**3. No decision for a specific group**		13.3	19.1
**a. Incorrect allocation due to lack of control**	• "It is difficult to see who really has only one sexual partner or changing sexual partners [...] and those who can lie well may get away with it."	2.6	1.4
**b. Burden of responsibility / general decision not reasonable / no fair solution possible**	• "Experts such as doctors, researchers and politicians should develop a concept and then implement it."<br>• "I do not want to judge which person "deserves" the vaccination or not!"<br>• "In such a case, individual medically justified solutions should be sought. General decisions according to age or behaviour (?) are always doubtful and not justifiable."	10.7	17.7
**4. Other reason**		8.6	17.7

* A single statement could be assigned to several subcategories.
