# Supplementary material for: Providing laypeople with results from dynamic infectious disease modelling studies affects their allocation preference for scarce medical resources—a factorial experiment
Source: BMC Public Health. 2022 Mar 23;22:572. doi: 10.1186/s12889-022-13000-7 (PMC8940588; doi:10.1186/s12889-022-13000-7)
Supplement: Supplementary file 5 — Additional file 5. Relative frequencies (%) of choice of allocation scheme in the two scenarios stratified by the randomization factor “model-based information on expected population-level effects”. [file 12889_2022_13000_MOESM5_ESM.pdf]

Additional File 5 – Relative frequencies (%) of choice of allocation scheme in the two scenarios stratified by the randomization factor "model-based information on expected population-level effects"

	Prevention ($p < 0.001$)			Treatment ($p = 0.03$)		
	No info (n = 149)	Additional info: 10,000 deaths (n = 144)	Additional info: 20,000 deaths (n = 148)	No info (n = 144)	Additional info: 10,000 deaths (n = 146)	Additional info: 20,000 deaths (n = 147)
		p = 0.80 among participants who received any info			p = 0.76 among participants who received any info	
**Random allocation**	12.8	11.8	12.2	8.3	4.1	7.5
**First come, first served**				27.1	21.2	17.7
**Young individuals first**	22.1	6.2	8.8	11.1	8.9	7.5
**Promiscuous individuals first**	34.2	54.9	49.3	12.5	28.1	32.0
**Long-lasting partner-ships first**	16.8	11.1	10.1	9.7	10.3	8.8
**Undecided**	14.1	16.0	19.6	31.2	27.4	26.5
