# Supplementary material for: Providing laypeople with results from dynamic infectious disease modelling studies affects their allocation preference for scarce medical resources—a factorial experiment
Source: BMC Public Health. 2022 Mar 23;22:572. doi: 10.1186/s12889-022-13000-7 (PMC8940588; doi:10.1186/s12889-022-13000-7)

**Additional File 7 – Relative frequencies (%) of choice of allocation scheme in the two scenarios stratified by highest completed educational level and the randomization factor “model-based information on expected population-level effects”**

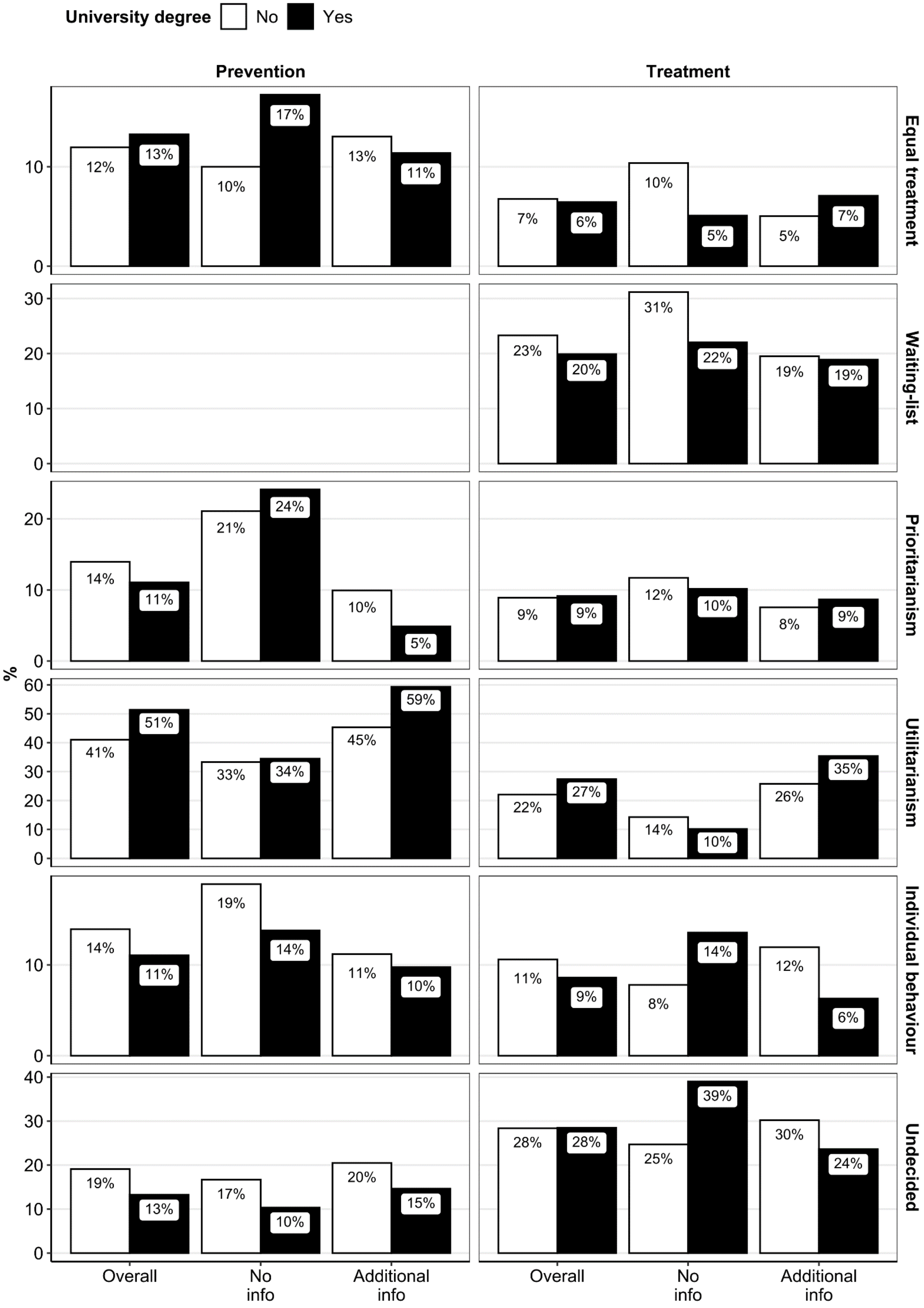

Supplement: Supplementary file 7 — Additional file 7. Relative frequencies (%) of choice of allocation scheme in the two scenarios stratified by highest completed educational level and the randomization factor “model-based information on expected population-level effects”. [file 12889_2022_13000_MOESM7_ESM.pdf]
